# Acetazolamide affects Aquaporin 1 expression of cultured choroid plexus cells

**DOI:** 10.1186/1743-8454-7-S1-S33

**Published:** 2010-12-15

**Authors:** Jogi V Pattisapu, Pouya Ameli, Meenu Madan, Amin Yu, Srinu Chigurupathi, Sic L Chan

**Affiliations:** 1 Burnett School of Biomedical Sciences, University of Central Florida College of Medicine Bldg 20, Room 335 4000 Central Florida Blvd Orlando FL 32816, USA

## Background

Acetazolamide (AZA), the only drug generally approved for hydrocephalus, is effective in only 25-30% of patients, and its effect on basal fluid flow in choroid plexus (CP) is unknown. The drug reversibly inhibits Aquaporin 4 (AQP4), the most highly expressed ‘water pore’ in the brain, and it is postulated that it reduces CSF production by modulating the expression and/or function of AQP1 (mostly found in the apical membrane of CP). In this study, we sought to elucidate the direct effect of AZA on basal fluid flow in CP.

## Materials and methods

CP tissues were harvested from 10-day Sprague-Dawley rat pups. CP cultures were grown to confluence in Transwell permeable supports and tested using: a) Kir 7.1 immunocytochemistry to confirm CP cell morphology; b) Lucifer Yellow assay and trans-epithelial electrical resistance (TEER) to determine level of confluence; c) fluid assays using TRITC-labeled Dextran to assay the direction and extent of fluid flow through the monolayers; d) expression of AQP1 protein by immunoblotting and immunocytochemistry.

## Results

Immunblotting and immunocytochemical analyses showed that AQP1 protein level decreases rapidly with 10 um AZA treatment. The reduction of AQP1 protein was transient as its level returned to baseline 12 hours after AZA exposure. Transwell fluid assay indicated that the early loss of AQP protein is correlated with decreased basal fluid flow in CP.

## Conclusions

The observed effect of AZA on AQP1 protein level suggests that AZA can directly modulate basal fluid flow in CP. The AZA induced effect in CP resulted in an initial fluid flow into the basolateral side of the transwells, with subsequent flow into the apical side of the CP membranes. Our results indicate that modulating AQP1 function via effectively targeted pharmaceuticals could potentially yield new treatments for hydrocephalus.

